# Off-label caplacizumab as add-on therapy in a 9-year-old boy with refractory aTTP

**DOI:** 10.1007/s00277-021-04740-4

**Published:** 2021-12-21

**Authors:** Marinella Veltroni, Francesco Pegoraro, Barbara Scappini, Francesca Brugnolo, Elisa Allegro, Stefano Ermini, Annalisa Tondo, Ilaria Fotzi, Franco Bambi, Claudio Favre

**Affiliations:** 1 Department of Pediatric Hematology/Oncology and HSCT, Meyer Children’s University Hospital, Viale G. Pieraccini, 24, 50139 Florence, Italy; 2 Department of Health Science, University of Firenze, Firenze, Italy; 3 Hematology Unit, Azienda Ospedaliero-Universitaria Careggi, Firenze, Italy; 4 Department of Experimental and Clinical Medicine, University of Firenze, Firenze, Italy; 5 Department of Immunohematology, Transfusion Medicine and Laboratory, Meyer Children’s University Hospital, Firenze, Italy

To the Editor,

Acquired thrombotic thrombocytopenic purpura (aTTP) is a rare but life-threatening immune-mediated disease caused by autoantibodies directed against the von Willebrand (vWF) cleaving protease ADAMTS13. The resulting severe deficiency of ADAMTS13 is responsible for a clinical picture that comprises hemolysis, thrombocytopenia, and widespread organ disfunction [1]. The traditional approach to aTTP used to combine plasma exchange (PEX) with immunosuppressants, but mortality rate remained remarkable (up to 10–15%) in refractory patients [2]. Caplacizumab is a humanized anti-vWF nanobody that proved effective and safe in adults [3]. Data on caplacizumab use in children, and specifically for those < 12 years, are anecdotic. [4]

**Fig. 1:**
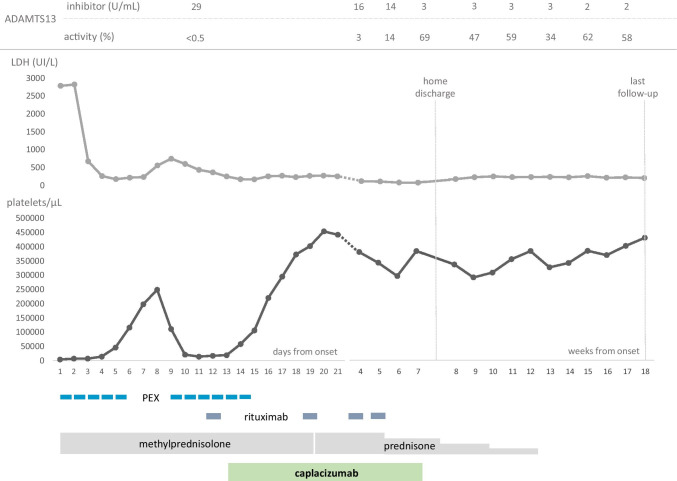
Visual summary including laboratory findings and treatment. Treatment: PEX daily, days 1–5 and 9–14; intravenous rituximab, 375 mg/m^2^ weekly, weeks 2–5; intravenous methylprednisone 1.5 mg/kg bid, days 1–19; oral prednisone 1 mg/kg bid for 18 days and then slowly tapered within 7 weeks; caplacizumab 10 mg daily, intravenous day 13 and subcutaneous days 14–42. LDH: lactate dehydrogenase; PEX: plasma exchange

A 9-year boy presented at the Emergency Department with rapid onset abdominal pain and macrohematuria. No significant history or recent infection was reported. At clinical examination, he had a widespread petechial rash and hypertension (135/90 mmHg). Laboratory studies showed a normal white blood cell count, low platelets (4 × 10^3^/µl) and hemoglobin (10.7 g/dL), 12% of schistocytes on blood smear, bilirubin 7 mg/dL, lactate dehydrogenase 2786 U/L, and undetectable haptoglobin. No additional evidence of central nervous system and kidney involvement (after resolution of hematuria) was detected. However, due to the severe hematologic involvement, the patient was transferred to the intensive care unit where he received intravenous steroids (prednisone equivalent dose of 2 mg/kg daily) and a 5-day course of PEX for presumed TTP, with good response in terms of platelet count (116 × 10^3^/µl at day 6) (Fig. 1). After remaining stable for 2 days, the platelet count rapidly decreased to 14 × 10^3^/µl, and PEX was started again. At the same time, the ADAMTS13 inhibitor was detected at 29 U/mL and ADAMTS13 activity suppressed (< 0.5%); therefore, a diagnosis of refractory aTTP was made, despite no trigger being identified. Considering the severity of the clinical course, off-label administration of caplacizumab was considered. After approval by local IRB and acquisition of parents’ consent, he received intravenous caplacizumab (at adult dose, 10 mg) followed by daily subcutaneous administrations at the same dosing for 30 days. Intravenous rituximab (375 mg/m^2^ weekly for four weeks) was also administered. A rapid and sustained response of platelet values was obtained (220 × 10^3^/µl after 3 days, Fig. 1). Treatment with caplacizumab was well tolerated, except for a mild cutaneous reaction in the sites of subcutaneous administration. The patient was finally discharged after 52 days in good clinical condition with a platelet count of 376 × 10^3^/µl and an ADAMTS13 activity of 69%. The patient later underwent weekly follow-up evaluations, always remaining in good clinical condition and presenting normal platelets and ADAMTS13 activity (Fig. 1).

Caplacizumab has proven effective in adults as add-on therapy for refractory aTTP [3]. Limited real-life data and sporadic case-reports describe its use in children, [4, 5] but indications for caplacizumab administration are limited to patients older than 12 years. Off-label administration of caplacizumab proved effective and safe for refractory aTTP in our patient, in combination with PEX and immunosuppressive drugs. Despite limited evidence being available on caplacizumab efficacy and safety in younger children, its use in refractory aTTP is a valuable option for patients not responding or relapsing early on conventional therapies.
